# Compromise in Political Theory

**DOI:** 10.1177/14789299221131268

**Published:** 2023-01-04

**Authors:** Friderike Spang

**Affiliations:** Faculty of Law, Criminal Justice and Public Administration, University of Lausanne, Quartier UNIL-Chamberonne, Lausanne, Switzerland

**Keywords:** compromise, disagreement, pluralism, respect, deliberation, negotiation

## Abstract

This review article provides a topic-centered overview of the state of compromise in political theory, where compromise is increasingly discussed as a promising approach to dealing with disagreement in politics and society. Given the growing literature on compromise, a systematic approach to the topic is due. The first sections are focused on clarifying the concept of compromise, while the remainder of the article offers different perspectives on those aspects of compromise that are subject to debate.

## Introduction

In its most basic sense, a compromise can be understood as a form of agreement that has the purpose of accommodating conflicting views or claims.^1^ As such, compromise is increasingly discussed in political theory as a legitimate approach to dealing with disagreement in politics and society (Baume and Novak, 2020b; Bellamy, 1999, 2012; Gutmann and Thompson, 2012; Mansbridge et al., 2010; May, 2005; Rostbøll and Scavenius, 2019; Warren and Mansbridge, 2016; Weinstock, 2006, 2013).^2^ But aside from a wide acknowledgment that compromise can constitute a legitimate approach to dealing with disagreement, the literature on compromise is characterized by significant differences and controversies—which is not surprising, given the complexity inherent in the subject of compromise. This review article aims to shed light on the complexity of compromise first by clarifying central features of compromise and second by identifying major issues of dispute.^3^

The article starts by distinguishing compromise from consensus, which simultaneously serves the purpose of presenting those features of compromise that characterize it as a distinct concept. The second section depicts different forms that a compromise can take, thus further expanding on the distinctive features of compromise. Next, the article addresses the question of whether compromise occurs primarily between different persons or whether a person also has to compromise with herself to reach a compromise with others. The fourth section deals with the process of achieving a compromise, thereby portraying a development in the literature from negotiation to more deliberative processes. The fifth section surveys different views on the controversial question of why we should compromise, including the question of whether compromise is justified for principled or for pragmatic reasons. In the sixth section, the article illustrates different approaches to the limits of compromise, that is, to the question of when a compromise is *not* justified. The last section outlines areas for future research.

## Compromise Versus Consensus

This section distinguishes compromise from consensus. It is important to distinguish between both concepts because they have significant features in common and are therefore easily confused. Like consensus, compromise is a possible response to disagreement or conflict. And like consensus, compromise can be placed on the “solution side” within the spectrum of possible responses to disagreement. As potential solutions to disagreement, both compromise and consensus differ from responses that maintain the status quo of disagreement or conflict (Bellamy et al., 2012).

But even though compromise and consensus both constitute potential solutions to disagreement, they are not the same kind of solution. Unlike compromise, consensus requires the parties to a disagreement to change their minds on the controversial issue. If a consensus is achieved, this means that the disagreeing parties consider the agreement to be better than (or at least as good as) their initial positions (Bellamy et al., 2012; Weinstock, 2006, 2013). Compromise, in contrast, is characterized by the fact that disagreeing parties hold on to their opposing views. As Daniel Weinstock (2013: 540) puts it, “it does not count as a compromise when you change your mind.” In a compromise, disagreeing parties *agree to* partially concede their claims to the demands of the other party, but they do not *agree with* the other party’s demands.^4^

It can therefore be said that consensus resolves disagreement in an *epistemic* sense, while compromise resolves disagreement in a *practical* sense, that is, by preventing negative consequences of continued disagreement. As Manon Westphal (2019) points out, compromises *settle* a given conflict but do not *resolve* the underlying disagreements. But even though the parties to a compromise continue disagreeing on a controversial issue, a compromise can prevent their disagreement from spiraling into a full-blown conflict.

Compromise also differs from consensus in that the former characteristically involves a sense of regret or dissatisfaction (Lepora, 2012; Lepora and Goodin, 2013). Since the parties to a compromise continue to believe that they are right and that the other party is wrong, agreeing on a compromise means agreeing to a solution that will partially realize values that one considers to be wrong. This is especially true for compromises on emotional issues, as is typically the case for issues of moral or political significance. Consensus, in contrast, does not involve regret or dissatisfaction. On the contrary, consensus (at least theoretically) leaves all parties satisfied, since all parties consider their consensual arrangement to be superior to (or equally good as) their original point of view.

Some scholars therefore claim that consensus is normatively superior to compromise. Amy Gutmann and Dennis Thompson (2012: 13), for example, state that “few doubt that consensus is desirable if it can be found, and most agree that it is usually preferable to the standard form of compromise, which leaves all parties dissatisfied.” Similarly, Philippe Van Parijs (2012: 480) claims that “even the best compromise (. . .) is still not quite as good as an (unconstrained) consensus.”

Yet, even though consensus may be generally more desirable than compromise, compromise can be more desirable under certain circumstances. More specifically, compromise can be more desirable than consensus because it is more feasible when it comes to dealing with disagreement in real life (Bellamy et al., 2012; Gutmann and Thompson, 2004, 2012; Westphal, 2019). Furthermore, it has been pointed out that compromise is not only more realistic than consensus but it can also enhance creativity and problem-solving capacities (Al Ramiah and Hewstone, 2012).

In addition to the argument from feasibility, compromise has been endorsed as a desirable response to what John Rawls (2001: 4) has called “the fact of reasonable pluralism.”^5^ The argument here is that compromise, but not consensus, can accommodate reasonable disagreements that are inevitably part of pluralistic societies. More specifically, in the case of reasonable disagreement, compromise, but not consensus, allows for equal concern and respect for the reasonable views that are in conflict (Bellamy et al., 2012). In addition, it can be argued that if parties to a disagreement have equally reasonable (but irreconcilable) views, consensus is not desirable, because it requires an unjustifiable change of mind from those who hold reasonable views. Compromise, in contrast, allows disagreeing parties to hold on to their reasonable views, thus constituting a more desirable solution to reasonable disagreement.^6^

## Different Kinds of Compromise

An undisputed feature of compromise is that it consists of mutual and voluntary concessions (Bellamy et al., 2012; Bohman, 1996; Jones and O’Flynn, 2012; Lepora, 2012; Lepora and Goodin, 2013; Margalit, 2010; May, 2013). As Van Parijs (2012: 469) points out “a compromise is an agreement, but not just any agreement. Its distinctiveness resides in the mutual concessions involved.” Concessions can, however, be of different kinds, leading to different kinds of compromise. More concretely, depending on the kind of concessions on which a compromise is based, we can distinguish between three kinds of compromise: intersection compromise, conjunction compromise, and substitution compromise (Lepora, 2012; Lepora and Goodin, 2013).

Intersection compromise is based on overlapping principles, while controversial principles are excluded from the agreement.^7^ Intersection compromise therefore applies to cases of disagreement where the involved parties have partially overlapping principles. For example, if party P1 endorses principles A, B, and C, and party P2 endorses principles A, D, and E, an intersection compromise would be based exclusively on principle A, while principles B, C, D, and E would be excluded from the compromise agreement (Lepora, 2012; Lepora and Goodin, 2013).

Disagreeing parties might, however, not be willing (or able) to base an agreement exclusively on shared principles; or they might simply not share relevant principles in the first place. In such cases, where no intersection compromise is possible, two other kinds of compromise are available. Both will be illustrated with reference to the following example: Valerie is a committed vegan for ethical reasons. She plans to have brunch with her non-vegan friend Nancy. Nancy desires to eat scrambled eggs (E) with bacon (B), while Valerie desires a plant-based breakfast (P) in a vegan location (L). In this case, Valerie and Nancy do not have overlapping principles on which they could base an intersection compromise. Nancy’s principles (E, B) and Valerie’s principles (P, L) are mutually exclusive: you cannot have eggs and bacon in a vegan restaurant. Two kinds of compromise are available in this case.

One option is a conjunction compromise, which integrates some of the conflicting principles (Lepora, 2012; Lepora and Goodin, 2013).^8^ In the above example, a conjunction compromise could be a vegetarian (but not vegan) restaurant that offers vegan options. In this case, Nancy would be able to eat eggs, but she would have to refrain from eating bacon, while Valerie would have the option to eat vegan food, but in a non-vegan location. In this conjunction compromise, Valerie gets (P) while sacrificing (L), and Nancy gets (E) while sacrificing (B).

Another option to accommodate Valerie and Nancy’s disagreement is a substitution compromise. Substitution compromise consists only of principles that are not part of the original disagreement (Lepora, 2012; Lepora and Goodin, 2013)–it “changes the subject,” so to speak (Weinstock, 2013: 545). For example, a substitution compromise for Valerie and Nancy’s disagreement could be to skip brunch altogether and have a glass of wine in the evening instead. This solution still qualifies as a compromise, since neither party gets what they initially wanted; and it qualifies as a *substitution* compromise because it does not involve the conflicting principles (i.e. principles (P) and (L) for Valerie, and principles (E) and (B) for Nancy).

Another important distinction is between compromise that accommodates moral disagreement (“moral compromise”) and compromise that accommodates non-moral disagreement (“non-moral compromise”). The conceptual distinction between principles and interests serves as a useful framework for addressing the difference between compromises on moral and non-moral disagreements. Moral compromise can be understood to involve principles, that is, beliefs and values that are based on moral convictions and that are often part of one’s identity. Non-moral compromise, in contrast, pertains to mere interests, including material interests such as income and wealth (Gutmann and Thompson, 2012).

Regarding moral compromise, the question arises whether it is realistic to expect disagreeing parties to partially concede what they consider to be morally right, and to partially accept what they consider to be morally wrong. Furthermore, as Lepora emphasizes, not only does a moral compromise require us to partially accept what we consider to be wrong in theory, but it also requires us to partake in (what we consider to be) wrongdoing in practice. Therefore, as Chiara Lepora (2012: 2) puts it, “from each party’s perspective, compromise necessarily involves interacting with, and sometimes contributing to, wrongdoing.” Moreover, Theodore Benditt points out that moral compromise can negatively affect one’s sense of self. If we accept a moral compromise, we might lose esteem not only for the other party but also for ourselves (Benditt, 1979)—we are compromised, as the saying goes.

In contrast to moral compromise, the realization of non-moral compromise is usually less problematic, because mere interests tend to be less attached to our sense of integrity, our conception of right and wrong, and they also tend to be less emotionally salient. Indeed, according to Benditt (1979: 27–28), “it is much easier to accept a compromise between competing interests—particularly when they are expressible in terms of a numerical scale like money—than between opposed principles which purport to be objectively valid.”

One might object that the very distinction between principles and interests is problematic, especially in the political sphere.^9^Alin Fumurescu (2013: 19), for example, considers this distinction to be “an almost impossible endeavor” when it comes to politics, because crucial questions, such as budget allocations, can be portrayed as either principles or interests, depending on what is deemed more suitable. In a similar vein, Andrew Sabl (2018: 274) points out that “every non-trivial political question seems a matter of deep principle to some” and that the distinction between interests and principles is therefore hard to sustain in politics.

Admittedly, the distinction between principles and interests is fuzzy, especially in the political sphere, where, indeed, the question arises of whether there are ever non-moral compromises, that is, compromises that do not, in one way or another, involve principles that are based on moral convictions. While this may be true for political decision-making, strictly speaking (e.g. law-making), compromises more generally speaking (e.g. between citizens) can and should be classified along categories of moral versus non-moral concerns. Otherwise, if there is no distinction between moral and non-moral compromises, we are bound to assume that there is no significant difference between a compromise on, say, the price for a pound of apples and abortion rights. As Benditt (1979: 32) remarks, however we draw the difference between principles and interests, the point remains that “there is undoubtedly a difference in the character of the conflict when principles and ideals are explicitly involved as opposed to when the parties see the conflict as between interests.” Therefore, while the distinction between principles and interests may be hard to sustain at the political level, it may still apply to compromises in general—even though it is, perhaps, less a question of “either-or,” in the sense that a compromise belongs to either the moral or non-moral domain; and more a question of degree, in the sense that a compromise should be located along a *spectrum* of moral and less moral matters.

## Who Compromises?

Compromise can be conceived of in an *interpersonal* and *intrapersonal* sense. In the interpersonal sense, compromise occurs between different persons who participate in a decision-making process (May, 2013). As such, a compromise can be struck between individual citizens or between their representatives, for example, legislators or lawyers (Jones and O’Flynn, 2012).^10^ In the intrapersonal sense, compromise occurs within a person’s head. This conception of compromise refers to the fact that a person also must compromise with herself if she is to compromise with another person.

Peter Jones and Ian O’Flynn claim that the standard meaning of compromise refers to the interpersonal sense. The authors argue that the intrapersonal notion of compromise is “figurative and parasitic upon the standard notion of compromise as an interpersonal or inter-party matter” (Jones and O’Flynn, 2012: 118). Similarly, Simon May (2013) holds that compromise is essentially an interpersonal matter.

In contrast, Lepora (2012) argues that intrapersonal compromise is, in fact, the more fundamental of both conceptions and logically prior to interpersonal compromise. According to her conception of intrapersonal compromise, a person compromises with herself if she sacrifices principles that conflict with other principles that she endorses: “in the intra-personal case, the compromise is among principles all of which you harbour but not all of which can be simultaneously pursued” (Lepora, 2012: 3). Thus understood, intrapersonal compromise consists in a decision on which of the conflicting principles we are willing to sacrifice, and it therefore logically precedes the possibility of achieving an interpersonal compromise: interpersonal compromise requires first an internal compromise between our own principles (Lepora, 2012).

Taking Lepora’s argument a step further, we might consider that intrapersonal compromise can entail the decision to compromise in the first place. This would be the case if, for example, a person’s core principle is to never give up on his ideals—in other words, not to compromise on his values. In this case, the decision to compromise is itself a compromise, because the person decides to compromise on his core principle not to compromise.

## How to Compromise?

Traditionally, compromise is considered to be the outcome of negotiation processes during which the involved parties bargain for the best possible outcome. In contrast, deliberative processes during which the participants exchange reasons for their respective views are deemed appropriate only for achieving consensus. This traditional view is deeply anchored in everyday language, where compromise is often tied to negotiation or bargaining. Furthermore, this view is also represented in the academic literature, for example, when Van Parijs (2012: 469) states that “*negotiation* can lead to a compromise that avoids the costs and risks of conflict, exit or arbitration, whereas *deliberation* can lead to a consensus about what is required for a fair solution” (emphasis in original). Similarly, May (2018: 150) ties compromise to negotiation by emphasizing that “compromise is the paradigmatic feature of negotiation.” From this perspective, the idea is that we can achieve a compromise by negotiating or bargaining with our opponents, while deliberation is only required if we aim for a consensus.

Challenging the traditional “division of labor” between compromise- and consensus-building processes, scholars of deliberative democracy increasingly emphasize a connection between compromise and deliberation. Gutmann and Thompson (2004: 12), for example, suggest that without deliberation, “we forsake the possibility of arriving at a genuine moral compromise.” Similarly, Weinstock (2013: 540) argues that “the attempt to arrive at a compromise is an exercise in moral deliberation rather than a simple exercise of ‘horse trading’” and that, in the context of pluralist societies, “compromise should be the goal that political deliberation sets for itself” (Weinstock, 2017: 636). Moreover, Mark Warren and Jane Mansbridge (2016) conceive of compromise as a possible outcome of deliberation as well as “deliberative negotiation”, that is, of negotiation processes that are characterized by deliberative features.

Furthermore, focusing on fair compromise specifically, Peter Jones and Ian O’Flynn (2012: 127) state that “a substantively fair compromise is more likely to arise if the compromising process takes the form of deliberation rather than bargaining.” Also addressing fair compromise, Friderike Spang (2021) takes this claim a step further and argues that deliberation is a *structural* necessity for achieving a fair compromise and that it would be counterproductive to seek a fair compromise through bargaining or negotiating. Moreover, Michele Moody-Adams (2018: 191) contends that (principled) compromise “inescapably embodies deliberative ideals such as fairness, mutual respect, and equality of opportunity to influence outcomes” and that such compromises should be integrated into deliberative processes. Given the mounting work on the connections between compromise and deliberation, it stands to reason that the question of “how to compromise” will increasingly shift into the deliberative terrain.

In contrast to these works, Eric Beerbohm (2018) critiques that deliberative conceptions of compromise are “overly rigoristic” in their condemnation of both misrepresentation and threats. He argues that a prohibition of misrepresentation is unwarranted, since an “asymmetric” distribution of information is part of the legislature and it is not clear why one should correct misconceived ideas: “If, in a compromise setting, my preferred concession seems significant to another party because of their ignorance, why should I have to inform them otherwise?” (Beerbohm, 2018: 18). Furthermore, he claims that a requirement to avoid bluffing and threats and to sincerely disclose one’s position may not constitute a sustainable practice for reaching legislative compromise. Instead, Beerbohm argues, the practice of compromise must have room for “sharp dealing” with those that resort to bluffs or threats, thus employing tactics that would otherwise not be considered appropriate.

While adding a new perspective on potential shortcomings of more deliberative accounts of compromise, especially in the context of legislation, Beerbohm’s approach remains short on some accounts: It does not, for example, address the question of fairness: Can sharp dealing allow to reach a fair compromise? If so, how? Moreover, can sharp dealing accommodate minority positions when these seek to rectify an injustice? Furthermore, as Beerbohm (2018: 44) points out himself, his account of compromise has been developed “from the ground-up, from within a more empirically based picture of what parties to compromise see themselves as doing.” His approach thus also raises a larger, yet unanswered methodological question of whether theories of compromise should be conceived of from an ideal or non-ideal perspective (or both).

## Why Compromise?

The question “why compromise?” can be understood in two different ways. First, it can refer to the question of why someone would want to compromise, motivationally speaking.^11^ Second, it can refer to the normative question of why, or for which reasons, we should compromise. This section addresses the question in the latter, normative sense.

A prominent debate in political theory revolves around the question of whether compromise is justified for pragmatic or principled reasons. We have pragmatic reasons to compromise if we prefer a specific compromise over the alternative of not compromising regarding the consequences that either option yields. That is, if we agree to a compromise for pragmatic reasons, we assume that the compromise is necessary to achieve goals that are important to us (May, 2005) and that the compromise will improve (or at least not worsen) the status quo (May, 2013). In this context, May has advanced the influential argument that compromise is *only* justified for pragmatic reasons of this kind. As he puts it, “moral compromise in political life is only ever warranted for pragmatic reasons” (May, 2005: 317). By way of rejecting four arguments in favor of principled compromise (complexity, respect, accommodation, and reciprocity), May argues that moral compromises cannot be justified for other than pragmatic reasons, especially given the fact that moral compromises inevitably involve a moral loss for the compromising person.

Other theorists, in contrast, argue that compromise can also be justified for principled reasons. More specifically, while May *categorically* rejects compromise for principled reasons, supporters of principled compromise do not tend to categorically reject pragmatic compromise. Rather, they argue that compromise can *also* be justified for principled reasons. Weinstock (2013) distinctly challenges May’s refutation of principled compromise by defending four arguments in favor of principled compromise: The “argument from political contingency” holds that principled compromise can constitute a remedy for unavoidable democratic deficits in securing equal respect and inclusion; the “argument from embeddedness” states that we have a principled reason to compromise if we reject the idea of a “winner-takes-all” society, because compromise allows us to incorporate the concerns of others, even if doing so is not necessary for pragmatic reasons; “the argument from principled consequentialism” proceeds from the idea that “it is sometimes justified to take a consequentialist attitude toward the moral principles that we affirm” (Weinstock 2013: 553), so that a compromise can be justified by consequentialist considerations regarding real-world conditions for the realization of our moral principles; and finally, the “argument from epistemic finitude” advances the idea that if we acknowledge the limits of our reasoning capacities, we have a principled reason to compromise, especially if we disagree with persons that we consider to be epistemic peers. In a similar line of thought, addressing the question of how we should deal with deep disagreements in pluralistic democracies, Federico Zuolo and Giulia Bistagnino (2018) argue that the recognition of epistemic parity may provide a principled reason for seeking compromise.

Other authors refer to respect as a principled reason for compromise by emphasizing that in situations of reasonable disagreement, where conflicting principles are equally reasonable and at the same time irreconcilable, compromise can express respect for the diversity of beliefs and values that characterize pluralistic societies (Bellamy, 1999, 2012; Bellamy et al., 2012; Dobel, 1990). Moreover, pluralism itself has been invoked as a justification for compromise because compromise “is a kind of agreement *that does not deny the plurality of society*” (Bellamy et al., 2012: 279, italics in original). Westphal (2019) argues in this context that compromise in pluralistic democracies is both normatively desirable and feasible: It is feasible because it does not require the conflicting parties to resolve their (reasonable) disagreement, and it is normatively desirable because it can enable co-authorship of the political rules that shape the societies in which they live. Furthermore, Moody-Adams (2018: 197) points out that democratic ideals require “meaningful respect for the deliverances of individual conscience, along with robust tolerance of at least *some* of the political conflicts they may produce.”^12^ To accommodate resulting conflicts in a cooperative manner, she argues, we need to adopt an attitude that is favorable to principled compromise.

In contrast to these accounts, Fumurescu (2013: 41) advances a critical view on principled compromise: He shares John Morley’s concern that by elevating compromise from a political method of last resort to a democratic principle, it has “become an end in itself” and is therefore hard to constrain. Anton Ford (2018: 71) provides another critical perspective on principled compromise, focusing on its logic “that it is sometimes good to offer up concessions even though one is not forced to do so.” Ford acknowledges that this can be a virtuous trait—but only if we conceive of compromise in the standard *bipolar* sense, that is, focusing on the parties concluding the compromise. However, if we conceive of compromise in a *tripolar* sense, that is, including third parties that are *affected* by a compromise, things look differently. Indeed, Ford (2018: 72) argues that from the perspective of those at “the receiving end of injustice,” it would be absurd to choose someone as your representative who compromises as a matter of principle, because principled compromisers “will freely and voluntarily throw their own weight behind the injustice to which you are exposed.” This is problematic not only because the concessions contributing to injustice are not necessary, but also because the act of agreeing to unforced concessions adds further injustice to the affected third parties: “There is, after all, a political agent who is freely and knowingly lending its support to a measure that does them wrong” (Ford, 2018: 72).

Yet there may be scenarios where principled compromise works in favor of those at the receiving end of injustice. It is, for example, conceivable that principled compromisers are more likely to develop trusting relationships with their interlocutors over time, thus achieving perhaps more favorable outcomes for affected third parties in the long run than pragmatic compromisers. While the precise consequences of the tripolar conception for principled (and pragmatic) compromise thus require further clarification, Ford’s conception of compromise as a “tripolar affair” constitutes a crucial contribution to our understanding of compromise. Not only does it allow for a more encompassing evaluation of political compromises and related questions of representation, but it can also serve as a critical framework for assessing compromises made on behalf of those without a proper political voice.

## Limits of Compromise

While compromise can be normatively desirable (whether for pragmatic or principled reasons), it is also clear that a compromise is not always justified. But what precisely are the limits of compromise? Under which circumstances is a compromise not justifiable?

In his essay “On Compromise,” Morley specifically sets out to address the limits of compromise. He distinguishes in this context between legitimate and illegitimate compromises, each of which relates to a different attitude. Compromise is legitimate if we remain true to our views while not enforcing them on others. A legitimate compromiser therefore displays the following mindset: “I do not expect you to execute this improvement, or to surrender that prejudice, in my time. But at any rate it shall not be my fault if the improvement remains unknown or rejected.” In contrast, compromise is illegitimate if we pretend to accept what we consider to be untrue. Illegitimate compromise is therefore based on the following mindset: “I cannot persuade you to accept my truth; therefore I will pretend to accept your falsehood” (Morley, 2004 [1891]: 209).

More recently, Avishai Margalit’s (2010) “On compromise and rotten compromise” also discusses the question of limits. Margalit argues that “rotten compromises,” that is, agreements to establish inhumane regimes which exert humiliation and cruelty, are never justified—not even for the sake of securing international peace. According to Margalit, inhumane regimes are never justified because they erode morality and thereby the foundation of treating one another as fellow human beings. But he also states that rotten compromise is the only kind of compromise that warrants a categorical prohibition: “Only rotten compromises are bad enough to be avoided at all costs” (Margalit, 2010: 160). Indeed, Margalit emphasizes that even morally questionable compromises (except for rotten compromises) are often better than the alternative of not compromising, especially so if a compromise can secure peace.^13^

While in Margalit’s account a categorical rejection of compromise is restricted only to the case of rotten compromises, other theorists propose more narrow constraints. Weinstock (2013), for example, claims that we need to hold our ground against unreasonable persons.^14^ Similarly, Richard Bellamy (1999) argues that one should not compromise with those who put forward sexist or racist arguments, or with fanatics who are not willing to justify their views and who do not respect the opinion of others.

Gutmann and Thompson (2012) also invoke disrespect as a potential reason for refusing a compromise. The authors suggest that signs of disrespect, such as threats or manipulation, can warrant denying a compromise, even if that compromise would improve the status quo. However, Gutmann and Thompson also caution against the ambition to devise general criteria for differentiating between desirable and non-desirable compromise. In their view, “it is a mistake to try to find unconditional principles that separate acceptable from unacceptable compromises” (Gutmann and Thompson 2012: 49–50). Instead, we are to consider the specifics of the disagreement in question to determine the justifiability of compromise. Margalit (2010) similarly urges a case-by-case evaluation of the merit of concrete compromises, emphasizing that abstract rules cannot cover all possible scenarios in which a normative evaluation of compromise is necessary. A similar argument is advanced by Benditt (1979), who emphasizes that the limits of compromise cannot be determined in advance by abstract criteria—an unfortunate situation that, as he claims, often leaves us in a quandary.

Sabl (2018: 262) approaches the question of limits from yet a different angle by focusing less on “the dispositions of those contemplating compromise” and more on “the circumstances in which they find themselves.” Concretely, he proposes *necessity* as a normative standard for distinguishing between warranted from unforgivable refusals to compromise. Necessity, in Sabl’s (2018: 260) account, is related to the prevention of public harm: “Compromise is necessary when inaction by a certain deadline would cause either *diffuse but substantial and pervasive harm* to the citizenry as a whole, or *acute and dangerous harm* to a subset of it” (emphasis added). *Diffuse but substantial and pervasive harm* includes “irreversible damage to the provision of an uncontroversial public good” (Sabl, 2018: 260), such as police, prisons, and so on. In contrast, *acute and dangerous harm* pertains to essential welfare benefits such as subsistence aid or emergency medical care. Refusal to compromise, then, is warranted only if it does not lead to public harm understood in these both senses.

Finally, Matthijs Bogaards’s recent work on militant consociational democracy in Belgium provides us with a distinctly political application of the limits to compromise.^15^ As a militant consociational democracy, the Belgian political system refuses cooperation, including compromise, with extremist parties on both the right and the left. As Bogaards (2020: 194) puts it, “militant consociational democracy limits the possibility to compromise to a certain category of parties, those that are deemed liberally democratic.” These limits to compromise raise questions about the standards of both excluding and including the extreme right. While inclusion is problematic if a party is deemed an enemy of liberal democracy, exclusion clashes with the deliberative democratic ideal of listening to the other side—and indeed, in Belgium, “the other side” has not yet included extreme right voters. Bogaards therefore suggests using citizen deliberation—which has been successfully employed for bridging linguistic divides—to build bridges between extreme right and mainstream parties, thus also widening the scope for political compromises.

## Outlook

The subject of consociational democracy points to further research questions related to compromise.^16^ In a nutshell, consociational democracies are characterized by non-majoritarian power distribution, where all relevant segments of society (along socio-economic as well as territorial and cultural cleavages) are represented by elites, “who have to act as prudent leaders” (Sinardet, 2010: 349). As such, consociationalism can serve to manage conflicts in divided societies (Sinardet et al., 2010) and it was indeed developed by Arend Lijphart precisely “as a theory of political stability in plural societies” (Bogaards, 2020: 177; see also Deschouwer, 2006).

Belgium is a classic example for consociational democracy, also given the central place of compromise for accommodating the linguistic cleavage between the Dutch- and French-speaking population (Bogaards, 2020). However, the precise role of compromise in consociational democracies, and especially its role as distinct from consensus, requires further clarification. For example, Kris Deschouwer (2006: 904) emphasizes that the “fluctuation between conflict and compromise is a fundamental characteristic of the Belgian consociational democracy” and that the elites’ reliance on complex compromises explains successful conflict management in Belgium.^17^ In contrast, Dave Sinardet (2010: 348) emphasizes that “decision-making by consensus” is at the core of Belgian consociationalism, and that conflict regulation is “based on consensus between party elites” (Sinardet, 2010: 355).^18^ Given the centrality that is attributed to both compromise and consensus, future research should clarify their respective roles in the context of consociational democracies.

Another area of further research pertains to distinctly political compromises. While this survey has addressed compromises broadly conceived—covering compromises between individuals, groups, legislators, and so on—more work is needed on the specifics of compromises in politics, starting with the question of what political compromises are. As Ford (2018: 61) points out, there is some ambiguity in the concept of a political decision—such as a compromise—which might be understood “to cover any decision, on any question, made by any political agent” and political agents, in turn, can be understood to include “political parties, activist organizations, trade unions, public officials, and private citizens.” The question therefore arises of what, exactly, makes a compromise political and who are the relevant actors. Furthermore, does political compromise take a distinct shape in the case of *intra*-personal compromise? For example, in the context of law-making, a politician must balance not only between her own principles, but also between those of her constituents, her party, or national interests.^19^ Further research could clarify whether existing accounts of intrapersonal compromise sufficiently capture political compromises of this kind and if not, how such accounts might be conceptually expanded.

## Notes

1. Thus understood, the term “compromise” designates the outcome of an agreement-seeking process. It should be noted that “compromise” can also designate the agreement-seeking process itself (Golding, 1979; Lepora, 2012; Weinstock, 2013).

2. For a more critical assessment of the “uneasy relation between compromise and democracy,” especially regarding the relationship between compromise and publicity (understood as a value of good governance), see Baume and Novak (2020a).

3. Given the increasing number of publications on the subject of compromise and given the limited space available, this article does not aim to completely cover existing work on compromise, but rather to provide a survey of current debates within the field of political theory. For a broader perspective on objections to political compromise, see Baume and Papadopoulos (2022). For an in-depth historical account of compromise, see Fumurescu (2013). His “conceptual genealogy” reveals that different attitudes toward compromise until the late eighteenth century (negative in France and positive in England) have been intimately tied to diverging conceptions of both political- and self-representation.

4. May (2005) introduces a distinction between first- and second-order reasons: First-order reasons designate the reasons that we have for changing our minds on a controversial issue, while second-order reasons designate the reasons that we have for accepting a compromise.

5. The “fact of reasonable pluralism” refers to the idea that modern democratic societies are inherently characterized by a variety of religious, philosophical, and moral doctrines that can be equally reasonable and yet irreconcilable (Rawls, 2001). Note that Rawls (2001, 2005) prefers “overlapping consensus” to compromise, that is, an agreement that disagreeing parties can equally endorse, but for different reasons.

6. I elaborate on the aspect of pluralism in the Why compromise? section.

7. A principle is here understood to entail moral values rather than mere (monetary) interests.

8. Conjunction compromise has alternatively been called “integrative compromise” (Weinstock, 2013).

9. I thank one of the reviewers for raising this point.

10. While a compromise between legislators or lawyers has binding consequences, compromise between citizens serves the more informal purpose of ensuring continued cooperation in the midst of disagreement (see Bohman, 1996). Depending on how a compromise is reached, compromise between citizens can also have macro-political uptake—for example, if a compromise is reached through the venue of mini-publics. For an analysis of macro-political uptake through mini-publics, see Goodin and Dryzek (2006).

11. See, for example, Spang (forthcoming), who focuses on the role of emotions for our willingness to compromise on moral issues. Fumurescu (2013) argues that historically, the willingness to compromise in politics depended on prevailing conceptions of political- and self-representation.

12. Moody-Adams (2018: 190) conceives of meaningful respect as a form of respect that is “rooted in a critical and fundamentally non-relativist pluralism,” so that public respect may be refused for beliefs or ways of life that are “hostile to democratically legitimate purposes and institutions.”

13. Morally questionable (but not rotten) compromises are, for example, compromises that are based on suspicious motives (“shady deals”), that involve unfair exchanges (“shoddy deals”), or that exploit vulnerabilities (“shabby deals”). See Margalit (2010: 3–4).

14. Weinstock (2013) cautions, however, against the temptation to consider those who disagree with us to be unreasonable, simply *because* they disagree with us. Indeed, empirical evidence suggests that disagreeing parties tend to perceive each other as biased, a perception that can easily lead to conflict escalation (Kennedy and Pronin, 2008).

15. The concept of a militant democracy is based on the idea that democracies must defend themselves against their enemies (Bogaards, 2020). Bogaards considers Belgium to be a militant *liberal* democracy in the sense that the extreme-right party “Flemish Interest” is ostracized not because it is considered a threat to democracy, but because it is considered a threat to liberal values.

16. I thank one of the reviewers for drawing my attention to the relationship between compromise and consociationalism.

17. The reliance on compromise in Belgian politics also constitutes a “quite dramatic default option” because failure to compromise means failure to govern: “Either there is a compromise that is acceptable for both communities, or there is no longer any government” (Deschouwer, 2006: 905).

18. Further instances where Sinardet (2010: 349) mentions consensus rather than compromise: “Because society is divided, it is up to elites to reach consensus between its different composing elements” or “elites have to be prudent leaders pursuing consensus” (Sinardet, 2010: 353).

19. I owe this example to one of the reviewers.
